# Nonlinear Spike-And-Slab Sparse Coding for Interpretable Image Encoding

**DOI:** 10.1371/journal.pone.0124088

**Published:** 2015-05-08

**Authors:** Jacquelyn A. Shelton, Abdul-Saboor Sheikh, Jörg Bornschein, Philip Sterne, Jörg Lücke

**Affiliations:** 1 Department of Software Engineering and Theoretical Computer Science, Technical University Berlin, Berlin, Germany; 2 Department of Computer Science and Operations Research, University of Montreal, Montreal, Quebec, Canada; 3 Frankfurt Institute for Advanced Studies, Goethe-University Frankfurt, Frankfurt, Germany; 4 School of Medicine and Health Sciences and Cluster of Excellence Hearing4all, University of Oldenburg, Oldenburg, Germany; University of Verona, ITALY

## Abstract

Sparse coding is a popular approach to model natural images but has faced two main challenges: modelling low-level image components (such as edge-like structures and their occlusions) and modelling varying pixel intensities. Traditionally, images are modelled as a sparse linear superposition of dictionary elements, where the probabilistic view of this problem is that the coefficients follow a Laplace or Cauchy prior distribution. We propose a novel model that instead uses a *spike-and-slab prior* and *nonlinear combination of components*. With the prior, our model can easily represent exact zeros for e.g. the absence of an image component, such as an edge, and a distribution over non-zero pixel intensities. With the nonlinearity (the nonlinear max combination rule), the idea is to target occlusions; dictionary elements correspond to image components that can occlude each other. There are major consequences of the model assumptions made by both (non)linear approaches, thus the main goal of this paper is to isolate and highlight differences between them. Parameter optimization is analytically and computationally intractable in our model, thus as a main contribution we design an exact Gibbs sampler for efficient inference which we can apply to higher dimensional data using latent variable preselection. Results on natural and artificial occlusion-rich data with controlled forms of sparse structure show that our model can extract a sparse set of edge-like components that closely match the generating process, which we refer to as *interpretable* components. Furthermore, the sparseness of the solution closely follows the ground-truth number of components/edges in the images. The linear model did not learn such edge-like components with any level of sparsity. This suggests that our model can adaptively well-approximate and characterize the meaningful generation process.

## Introduction

Many natural signals, such as visual data, exist in a high-dimensional space. Understanding the structure of visual data is a challenging task that is often approached by forming parametric models of the data following some principles of optimality, in order to learn something about the data’s content and composition. As many signals have a low intrinsic dimensionality, in this paper we focus on the domain of *sparse coding models* to address the task of image modelling. The basic idea behind the sparsity principle is to represent a signal—such as an image—as a combination of few basis functions or features. With roots in signal processing, it is often thought that a model assuming or enforcing sparsity can recover the intrinsic signal dimensions and therefore better represent the relevant information content in the signal (e.g. [1, 2]). Furthermore, one would expect that if the algorithm learns meaningful hidden structure of the signal, then this approach would be successful at many data-driven tasks. When an algorithm can extract and represent the relevant information content from a signal that not only follows the generating process of that data but can also be easily interpreted in the context of the task at hand, we refer to this as *interpretable* data encoding.

Following early physiological recording studies [3] on simple cells in the visual cortex, sparse coding became popular as a model of the visual data encoding process in the mammalian primary visual cortex [4] and has now become not only the standard model to describe coding in simple cells, but also a very popular feature learning algorithm (e.g. [5, 6]). Formally, sparse coding (which will be referred to as ‘SC’) assumes that each image (also called an ‘observation’, or observed variables) **y** = (*y*_1_, …, *y*_*D*_)^*T*^ is associated with a sparse vector of latent variables **s** = (*s*_1_, …, *s*_*H*_)^*T*^ (also called latent ‘causes’ or coefficients of the data), where *D* and *H* denote the dimensionality of the observed image and the latent variable space, respectively. In the setting of visual data, the sparse latent vector **s** describes the set of the possible causes of an observed image and is associated with a set of image components, or *dictionary elements*, *W* ∈ ℝ^*D* × *H*^ (low-level image components, e.g. edge-like structures) where the absence of such an image component is associated with *s*_*h*_ = 0. In this way, sparsity means that most of the coefficients *s*_*h*_ in **s** are zero or close to zero.

The *standard linear sparse coding problem* is formulated as follows:

$$\begin{array}{c} loss\left(y^{\left(n\right)},W\right)\;\;\;:=\;\;\;\min_{s}\;\;\frac{1}{2}{\left|\right|y^{\left(n\right)}-Ws\left|\right|}_{2}^{2}\;+\;a{\left||s|\right|}_{1}, \end{array}$$ (1)

with the objective to minimize the loss between the image **y** ^(*n*)^ and its *linear* reconstruction/estimation **W** **s** (or equivalently ∑_*h*_ *s*_*h*_ **W**_*h*_ where **W** is the *D* × *H* matrix of **W**_*h*_ dictionary elements/components), with a penalty on the *l*_1_-norm of the vector **s**. The penalty is controlled by a regularization parameter *a*, which dictates how sparse the coefficients **s** in the reconstruction of **y** will be. Objective Eq (1) and associated optimization algorithms are also often referred to as *basis pursuit*[7] or the *Lasso*[8].

Probabilistically, linear SC can be formulated as a *generative model*:

$$\begin{array}{c} p\left(y\;|\;\Theta \right)=\int_{s}p\left(y\;|\;s,\Theta \right)\;p\left(s\;|\;\Theta \right)\;ds, \end{array}$$ (2)

where the latent causes are characterized by *p*(**s**∣Θ) with a sparse prior distribution. The observation/image described by *p*(**y**∣**s**, Θ) is typically a Gaussian distribution with a mean *μ* = ∑_*h*_ *s*_*h*_ **W**_*h*_, i.e. centered at the linear superposition of components **W**_*h*_ ∈ ℝ^*D*^. If the Laplace distribution is used as prior distribution, it can be shown that the minimization of objective Eq (1) with respect to the dictionary elements corresponds to expectation maximization (EM) learning using the maximum a-posteriori (MAP) approximation for the posterior (e.g. [9]). For dictionary learning, the formulation of objective Eq (1) is often the method of choice, and the focus is on efficient optimization of the dictionary. With these approaches, no prior parameters can be learned directly and the sparsity penalty, therefore, has to be set by hand or it has to be determined by cross-validation in another optimization loop. Furthermore, MAP estimates of the posterior can lead to a relatively coarse approximation, which has motivated improved probabilistic approaches for the standard model [10, 11].

**Fig 1:**
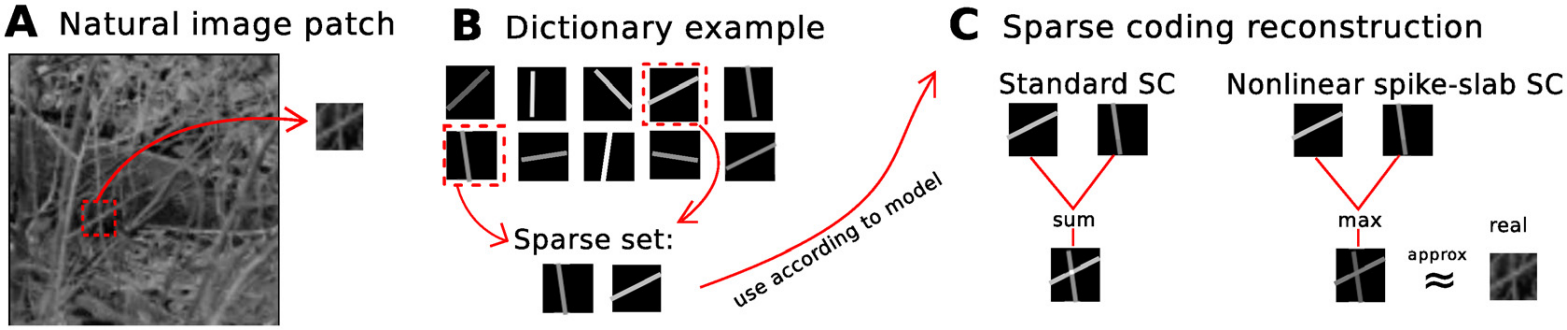
Toy example illustrating the problem setting: approximating occlusions in images. Given an image patch with occlusions (**A**), assume both the linear and nonlinear sparse coding models were given the true generating dictionary elements (**B**) and the task is for each model to use a sparse set of these to generate a reconstruction of the patch (**C**). **A** Example natural image with one patch to be reconstructed by the models. **B** 10 ground-truth dictionary elements, assumed to be known and with only 2 of 10 having generated the image patch. **C** Image reconstruction using the sparse dictionary set of the 2 models: the standard linear sparse coding model and the nonlinear spike-and-slab SC model. The linear sum leads to inaccurate pixel estimates when components overlap, whereas the nonlinear max aims to approximate this type of data more realistically in this scenario. Furthermore, the spike-and-slab prior (shown here for the the nonlinear model) allows the model to adapt the intensity of each image component to match what it observed in the data.

**Fig 2:**
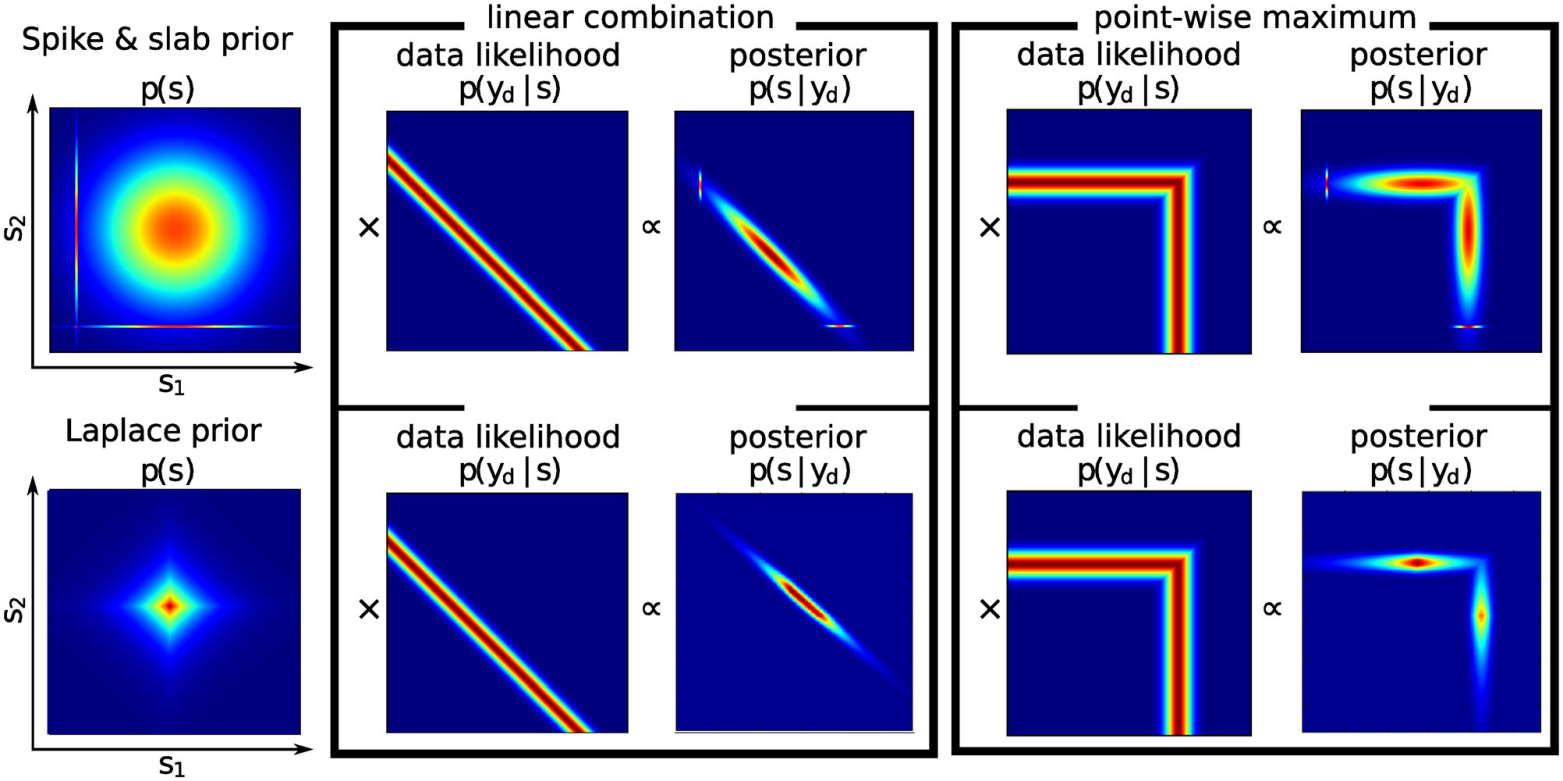
Illustration of choice of prior distribution and multimodality in the latent space. A H = 2-dimensional spike-and-slab and Laplace priors over latent variables and the multimodal posterior distribution induced by these priors for both linear and nonlinear data likelihoods.

The focus of this work is to investigate a new sparse coding model that forms a more realistic image model than the standard linear model with Laplace prior. After motivating and defining the model, we will systematically evaluate the differences to standard sparse coding. The problem setting we focus on is illustrated with the toy example in Fig 1. One can see that visual components (such as edges) are either present or absent (i.e. coefficient *s*_*h*_ = 0) in an image. This however points to the first challenge that standard models for sparse coding face: standard models using a Laplace or Cauchy prior distribution, which do not intrinsically represent exact zeros, can only either yield coefficients with exact zeros as an artifact of the optimization that artificially enforcing the coefficients to be zero (see e.g. [6, 11] for examples). These distributions are referred to as “weakly sparse”, as they have no coefficients actually at zero, but many very close to zero [12]. Other models, with use of a binary prior distribution, can represent exact zeros (to model e.g. the absence of a visual component with *s*_*h*_ = 0) without need for optimization techniques to induce them. These models cannot however model the range of intensities that the image components may manifest (e.g. when the component is present, it is represented by *s*_*h*_ = 1). An alternative and recently very popular prior is the spike-and-slab distribution (e.g. [12–15]), which is a distribution consisting of a discrete binary part and a continuous Gaussian part (see the first column in Fig 2 for an illustration of the spike-and-slab and Laplace priors). This prior can model not only the absence/presence of a component (via the binary ‘spike’) but also the visual intensity of that component (via the ‘slab’). Second, the standard model assumes that visual components linearly superimpose to form an image, although objects do not actually elicit summed intensity values when they happen to occlude each other. In this setting, when evaluating the pixel intensities of two overlapping components, the standard linear model would sum the two pixels, which poorly estimates the intensity, whereas the max infers that the pixel with the maximal intensity is occluding the other, offering a better estimate, illustrated in Fig 1C. Despite these two modelling caveats, the most work on SC models focuses on efficient inference of the optimal parameters for the linear model (e.g., [6, 11]) and not in assessing the model assumptions themselves. The standard linear model form offers mathematical convenience for inference, namely allowing the use of convex approaches (i.e. the posteriors over latent variables have only one mode, allowing for efficiency/accuracy of maximum a posteriori (MAP) estimations). Consequently, the standard model has continued to use a Laplace prior with a linear superposition, because changing the prior or changing the superposition assumption induces complex and multi modal posteriors and correspondingly poses a challenge for MAP estimates due to many locally optimal solutions. As a result, each proposed modification of the standard model has so far only been investigated in turn.

This work proposes a novel sparse coding model that combines both of these improvements – a *spike-and-slab distribution* and *nonlinear max combination of components* – in order to form a more realistic model of images. For our main technical contribution, we optimize our model by using a combined approximate inference approach with preselection of latent variables (for truncated approximate EM [16]) in combination with Gibbs sampling [17]. Importantly, as we expect to see the most salient differences between the models when occlusions are present, several sets of experiments focus on natural and artificial occlusion-rich datasets where we consider the task of dictionary learning and image reconstruction.

In our experiments we show that we can efficiently train this nonlinear model and perform inference assuming a reasonably high number of observed and latent variables. First, we show on artificial data that the method efficiently and accurately infers all model parameters, including data noise and sparsity. Next, we compare our nonlinear model to a state-of-the-art linear model on occlusion-rich datasets for the task of dictionary learning and image reconstruction on both artificial data with controlled forms of sparse structure as well as natural data. With experiments comparing the reconstruction of images by the two models, we show that the nonlinear model extracts/uses a sparse set of interpretable, holistic components that match the generating process, whereas the linear model (at all sparsity levels) uses components which are difficult to interpret and not aligned with the generating process. Finally, with experiments on image patches, we show that our model is consistent with *in vivo* neural recordings and learns image components with which linear models have struggled [18, 19]. With these data we also show that our model is consistent in the sense that the average posterior over the latent variables is approximately equal to the prior.

The paper is organized as follows: first, the proposed model will be presented, second, the details of the inference method will be described, third, all experimental results will be presented, and finally, the results will be discussed.

## Model: Nonlinear spike-and-slab sparse coding

We formulate the data generation process as the probabilistic generative model:

$$\begin{array}{c} p\left(y_{d}\;|\;s,\Theta \right)=𝓝\left(y_{d};\;\max_{h}\left\{s_{h}W_{dh}\right\},\;\sigma^{2}\right). \end{array}$$ (3)

Here, in contrast to the standard linear formulation in Eq (2), the likelihood contains the nonlinear term max_*h*_{*s*_*h*_ *W*_*dh*_} instead of the linear ∑_*h*_ *s*_*h*_ *W*_*dh*_ (the max_*h*_ which considers all *H* latent components and takes the *h* yielding the maximum value for *s*_*h*_ *W*_*dh*_). Also, the latent variable *s*_*h*_ is drawn from a spike-and-slab distribution given by *s*_*h*_ = *b*_*h*_ *z*_*h*_, where *b*_*h*_ is drawn from a Bernoulli distribution and *z*_*h*_ is drawn from a Gaussian distribution (ℬ and 𝓝, respectively), and is parameterized by:

$$\begin{array}{c} p\left(b_{h}\;|\;\Theta \right)=𝓑\left(b_{h};\pi \right)=\pi^{b_{h}}\;{\left(1-\pi \right)}^{1-b_{h}} \end{array}$$ (4)

$$\begin{array}{c} p\left(z_{h}\;|\;\Theta \right)=𝓝\left(z_{h};\;\mu_{\text{pr}},\sigma_{\text{pr}}^{2}\right), \end{array}$$ (5)

The columns of the matrix **W** = (*W*_*dh*_) are the dictionary elements/generative fields, ${\left({\text{W}}_{h}\right)}_{h=1}^{H}$, with one **W**_*h*_ associated with each latent variable *s*_*h*_. We denote the set of all parameters with Θ = (*π*, *μ*_pr_, *σ*_pr_, *W*, *σ*).

For inference and in order to optimize the parameters Θ of this model, we are interested in working with the posterior over the latent variables given by

$$\begin{array}{c} p\left(s|y,\theta \right)=\frac{p(y|s,\theta )\;\;p(s|\theta )}{\int_{s^{\prime }}p\left(y|s^{\prime },\theta \right)\;\;p\left(s^{\prime }|\theta \right)\;ds^{\prime }}. \end{array}$$ (6)

Identical to the standard sparse coding formulation in Eqs (1) and (2), our model assumes independent latent variables and Gaussian-distributed observations given the latent variables. In contrast to the standard formulation, the latents are not distributed according to a Laplace prior and the components (i.e. coefficients, dictionary elements, or generative fields) are not combined linearly. Fig 1 contains a toy illustration of part of the generative process and model differences between standard linear model and nonlinear model. Fig 1A shows an example natural image, eliciting naturally occurring occlusions of branches and twigs, from which a patch has been extracted in order to illustrate the effects of each model’s (non)linearity assumption. Fig 1B shows examples of how corresponding generating dictionary elements could look. For the sake of simplicity, this example does not incoorporate the learning process, and assumes each model is simply given these components and instructed which sparse set of components in 1**B** generated the image patch in 1**A**. Fig 1C shows how the (non)linear assumptions of the models manifest when the given components from 1**B** are combined according to each model in order to reconstruct the patch in 1**A**. As can be seen for the sum operation in 1**C**, standard linear sparse coding results in strong interference when the dictionary elements overlap, whereas the max can reconstruct the patch using one element or the other when the two overlap, thereby minimizing interference. This effect however leads to correlated multimodal posteriors since each observed pixel *y*_*d*_ must be explained by either one cause or the other, instead of the sum of both. An illustration of the posteriors of these models will be provided in the following section. This example suggests that the max can better model the occluding components (e.g. [19–21]). Furthermore, for simplification in this example, we implicitly forced all other dictionary elements in 1**B** to be unused, i.e. associated with coefficients of *s*_*h*_ = 0, which is only possible with a spike-and-slab prior (or other binary prior, which in turn, would not be able to incoorporate the various gray value intensities of the dictionary elements). Additionally, with the spike-and-slab prior (shown here for the nonlinear model in Fig 1C) allows the model to adapt the intensity of each image component used to match what it observed in the data. Please see [22] for a preliminary discussion of this model in a conference submission, in which a thorough analysis of the model was not provided and additionally it contained an error in the computation of expected values, which has been corrected here.

### Related Work

While work on improved optimization approaches for the standard sparse coding continues and is important for many applications, the above discussed limitations of the underlying generative data model have motivated a number of related studies on improved models. In recent years, spike-and-slab priors for linear models have frequently been used. The resulting challenges for parameter optimization have been addressed by applying factorized variational EM [13, 14, 23], truncated EM [15] or sampling [12]. Furthermore, the use of spike-and-slab priors aligns well with the goals of compressed sensing approaches [24]. In a standard formulation, an observed variable is re-expressed as a sum of bases where the corresponding coefficients have hard zeros, and correspondingly the objective function includes an ∣∣ ⋅ ∣∣_0_-norm instead of the ∣∣ ⋅ ∣∣_1_-norm seen in standard sparse coding (see e.g. [2] for a review).

Similarly, inference and learning for sparse coding models that replace the linear combination by nonlinear ones have been investigated. Hidden causes models with nonlinearly interacting signal sources include the noisy-or combination rule [25–30], exclusive causes [31] or a maximum superposition [19, 20, 32]. Also a combination of linear superposition followed by a sigmoidal nonlinearity (post-linear nonlinearities) have been investigated (nonlinear ICA [33], sigmoid belief networks [34]). By definition, noisy-or models and sigmoid belief networks assume hidden units and observed units to be binary, which generally entails different application domains than used for standard sparse coding. Furthermore, the implicit computational challenges have prevented a scaling to large numbers of hidden dimensions. Nonlinear ICA and models with maximum superposition can in principle assume continuous observed and hidden variables, and are consequently applicable to the same data domain as standard sparse coding. As for noisy-or models, nonlinear ICA is more challenging to scale to large hidden spaces, however. For the maximum nonlinearity, earlier models [32] focused on inference instead of unsupervised learning of model parameters. Recent approaches demonstrated scalability of sparse coding with maximum nonlinearity to large hidden and observe dimensions (Maximal causes analysis ‘MCA’, [19, 20]) but hidden variables were constrained to be binary in these cases. Binary priors avoid the analytical intractability usually resulting from continuous priors but they prevent a fine-tuned data representation and reconstruction with continuous coefficients.

We will return to these approaches in context of the results in the Discussion section.

## Methods

In this section we present the optimization of parameters in our model and the novel inference method developed to address the associated intractabilities.

### Parameter estimation

To estimate the model parameters Θ of the generative model in Eq (3) we use expectation maximization (EM). We do inference in the E-step with our proposed method combining sampling and latent preselection [17], which we will introduce in the next section. Optimization in the EM framework entails setting the free-energy to zero and solving for the model parameters (M-step equations) (e.g., [35]).

As an example we obtain the following formula for the estimate of image noise:

$$\begin{array}{c} {\hat{\sigma }}^{2}=\frac{1}{NDK}\sum_{n}\sum_{d}\sum_{k}{\left(\max_{h}\{W_{dh}s_{kh}^{\left(n\right)}\}-y_{d}^{\left(n\right)}\right)}^{2}, \end{array}$$ (7)

where we average over all *N* observed data points, *D* observed dimensions, and *K* Gibbs samples. However, this notation is rather unwieldy for a simple underlying idea. As such we will use the following notation:

$$\begin{array}{c} {\hat{\sigma }}^{2}={\left\langle W_{dh}s_{h}^{\left(n\right)}-y_{d}^{\left(n\right)}\right\rangle }^{*}, \end{array}$$ (8)

where we maximize for *h* and average over *n* and *d*. That is, we denote the expected values ⟨ . ⟩* to mean the following:

$$\begin{array}{c} {\left\langle f\left(s\right)\right\rangle }^{*}=\sum_{n}\frac{\int_{s}\;p\left(s|y^{\left(n\right)},\Theta \right)\;f\left(s\right)\;\delta \left(\text{h}\;\text{is}\;\text{max}\right)\;ds}{\int_{s}\;p\left(s|y^{\left(n\right)},\Theta \right)\;\delta \left(\text{h}\;\text{is}\;\text{max}\right)\;ds}, \end{array}$$ (9)

where *δ* is the indicator function denoting the domain to integrate over, namely where *h* is the maximum. See the S1 Derivation 1 for detailed derivation of update equations. Analogously, to compute the expectations of the Gaussian part of the prior distribution’s parameters, the mean ${\hat{\mu }}_{\text{pr}}$ and the noise ${\hat{\sigma }}_{\text{pr}}^{2}$, we denote ⟨ . ⟩** to mean the following:

$$\begin{array}{c} {\left\langle f\left(s\right)\right\rangle }^{**}=\sum_{n}\frac{\int_{s}\;p\left(s|y^{\left(n\right)},\Theta \right)\;f\left(s\right)\;\delta \left(s_{h}\neq 0\right)\;ds}{\int_{s}\;p\left(s|y^{\left(n\right)},\Theta \right)\;\delta \left(s_{h}\neq 0\right)\;ds}, \end{array}$$ (10)

which is identical to ⟨ . ⟩* in Eq (9) except that we are interested in support from *all* of the posterior distribution where *b*_*h*_ = 1, regardless of whether *s*_*h*_ is the maximal cause, and *δ* is modified accordingly.

Using the condensed notation in Eqs (9) and (10) allows us to concisely express the update equations for the remaining model parameters:

$$\begin{array}{c} {\hat{W}}_{hd}=\frac{{\left\langle s_{h}y_{d}\right\rangle }^{*}}{{\left\langle s_{h}^{2}\right\rangle }^{*}},\;\hat{\pi }=\left\langle \delta \left(s\right)\right\rangle , \end{array}$$ (11)

$$\begin{array}{c} {\hat{\mu }}_{\mathrm{pr}}={\left\langle s_{h}\right\rangle }^{**},\;{\hat{\sigma }}_{\mathrm{pr}}^{2}={\left\langle {\left(s_{h}-{\hat{\mu }}_{\mathrm{pr}}\right)}^{2}\right\rangle }^{**}\;\; \end{array}$$ (12)

In this model **W**_*h*_ can be scaled by an arbitrary factor *α* when the corresponding *s*_*h*_ is scaled by $\frac{1}{\alpha }$. To prevent **W** from becoming arbitrarily large (which would lead to arbitrarily small values of **s**), common practice is to constrain its columns (each latent cause) ${\left({\text{W}}_{h}\right)}_{h=1}^{H}$ to have an *l*_2_−norm less than or equal to one. Instead, we constrain all columns **W**_*h*_ to be equal to *D* (equivalent to normalizing expectation of *W*_*dh*_ to one, i.e. all entries are approximately equal to one). This normalization allows the ${\hat{\mu }}_{\text{pr}}$ to be intuitively more interpretable when comparing results on different datasets where the data dimensions *D* may vary.

As one can see in the above equations, in order to compute the parameter updates, we need to calculate several expectation values with respect to the posterior distribution. However, as mentioned in the introduction, the posterior distribution of a model (linear or nonlinear) with a spike-and-slab prior is strongly multimodal. See Fig 2 for illustration of the posteriors in the two dimensional case for both (non)linear models with spike-and-slab and Laplace priors. Calculating expectations of this posterior is intractable, thus we must develop a new inference method in order to cope with these computations.

### Inference: Exact Gibbs sampling with preselection of latents

As described, parameter optimization is very challenging in this model. Consequently, current inference methods cannot address the task. In order to efficiently handle the intractabilities and the complex posterior (multimodal, high dimensional) illustrated in Fig 2, we take a combined approximate inference approach [17]. Specifically we design and propose an exact Gibbs sampler for our model in order to draw samples from the unique form of our posterior after we have reduced the set of latent variables to those with the most posterior mass. Reduction via preselection is not strictly necessary, but significantly increases efficiency when considering high dimensional posteriors, particularly in sparse models. As such, we will first describe the sampling step and preselection only later.

**Gibbs Sampling.** Our main technical contribution for efficient inference in this model is an *exact Gibbs sampler for the multimodal posterior*. Previous work has used Gibbs sampling in combination with spike-and-slab models [36], and for increased efficiency in sparse Bayesian inference [37].

Our aim is to construct a Markov chain with the target density given by the conditional posterior distribution:

$$\begin{array}{c} p(s_{h}|s_{H\backslash h},y,\theta ) \end{array}$$ (13)

$$\begin{array}{c} \propto \;\;p\left(s_{h}|\theta \right)\;\;\prod_{d=1}^{D}p\left(y_{d}|s_{h},s_{H\backslash h},\theta \right). \end{array}$$ (14)

We see from Eq (14) that the distribution factorizes into *D*+1 factors: a *single factor* for the *prior* and **D* factors* for *each likelihood*.

As the difficult part to sample from is the likelihood, $\prod_{d=1}^{D}p(y_{d}|s_{h},{\text{s}}_{H\setminus h},\theta )$, where the nonlinearity of the max plays a role, we begin with its construction and only afterwards will we include the spike-and-slab prior. For the point-wise maximum nonlinear case we are considering, the likelihood of a single *D* dimension, *y*_*d*_, is a piecewise function defined as follows:

$$\begin{array}{c} p(y_{d}|s_{h},s_{H\backslash h},\theta ) \end{array}$$ (15)

$$\begin{array}{c} \;=𝓝(y_{d};\;\max_{h^{\prime }}\left\{W_{dh^{\prime }}s_{h^{\prime }}\right\},\sigma^{2}) \end{array}$$ (16)

$$\begin{array}{c} \;=\{\begin{array}{cc} \underset{\mathrm{constant}}{\underset{︸}{𝓝(y_{d};\;\underset{h^{\prime }\backslash h}{\max}\left\{W_{dh^{\prime }}s_{h^{\prime }}\right\},\sigma^{2})}} & \text{if}\;s_{h}<P_{d}\\ 𝓝(y_{d};\;W_{dh}s_{h},\sigma^{2}) & \text{if}\;s_{h}\geq P_{d}, \end{array} \end{array}$$ (17)

where the *transition point*, *P*_*d*_, is defined as the point where *s*_*h*_ *W*_*dh*_ becomes the maximal cause:

$$\begin{array}{c} \frac{P_{d}=\max_{h^{\prime }\in \left\{H\backslash h\right\}}\left\{W_{dh^{\prime }}s_{h^{\prime }}\right\}}{W_{dh}}. \end{array}$$ (18)

**Fig 3:**
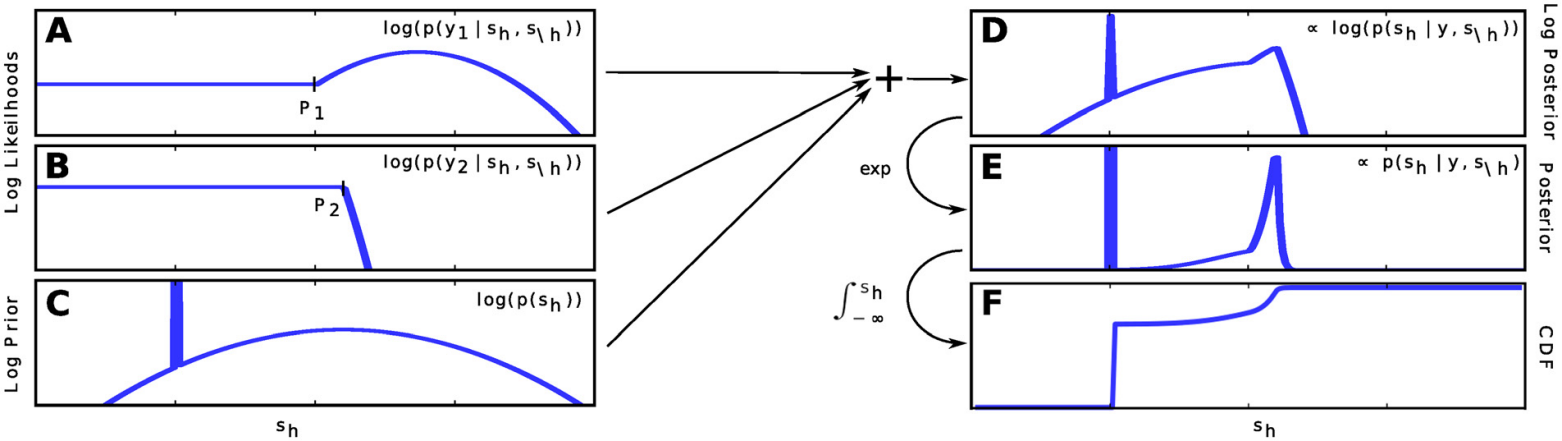
Construction of SSMCA-induced posterior for the Gibbs sampler. Left column: three contributing factors for the posterior ∝ *p*(*s*_*h*_ ∣ *s*_∖*h*_,**y**, Θ) in log space. **A** and **B**: Log likelihood functions each defined by a transition point *P*_*d*_ and left and right pieces *r*_*d*_(*s*_*h*_) and *l*_*d*_(*s*_*h*_). **C** Log prior, which consists of an overall Gaussian and the Dirac-peak at *s*_*h*_ = 0. **D** Log posterior, the sum of functions **A**, **B**, and **C** consists of *D* + 1 pieces plus the Dirac-peak at *s*_*h*_ = 0. **E** Exponentiation of the **D** log posterior. **F** CDF for *s*_*h*_ from which we do inverse transform sampling.

We refer to the two pieces of *y*_*d*_ in Eqs (15)–(17) as the *left* and *right* pieces of the function: *left*, *l*_*d*_(*s*_*h*_), when the latent cause is smaller than the transition point, *s*_*h*_ < *P*_*d*_, and *right*, *r*_*d*_(*s*_*h*_), when the latent is greater than or equal to the transition point, *s*_*h*_ ≥ *P*_*d*_. The left piece is constant with respect to *s*_*h*_ because the data is explained by another cause when the value of the latent *s*_*h*_ is smaller than the value of the transition point *P*_*d*_, and the right piece is a truncated Gaussian when considered a PDF of *s*_*h*_ (see Fig 3A–3B), because *s*_*h*_ is indeed explaining the data. Taking the logarithm of *p*(*y*_*d*_∣*s*_*h*_,**s**_*H*∖*h*_, *θ*) transforms Eq (15) into a left-piece constant and right-piece quadratic function. Expanding the expression for the logarithm of a given likelihood *p*(*y*_*d*_∣*s*_*h*_,**s**_*H*∖*h*_, *θ*), each left and right piece (the respective sides of each transition point *P*_*d*_) can be formulated as

$$\begin{array}{c} l_{d}\left(s_{h}\right)=-\frac{1}{2}\log\left(2\pi \right)-\log\left(\sigma \right)+\frac{1}{2\sigma^{2}}{\left(y_{d}-\max_{h^{\prime }\;\backslash h}\left\{W_{dh^{\prime }}s_{h}^{\prime }\right\}\right)}^{2} \end{array}$$ (19)

$$\begin{array}{c} r_{d}\left(s_{h}\right)=-\frac{1}{2}\log\left(2\pi \right)-\log\left(\sigma \right)+\frac{1}{2\sigma^{2}}{\left(y_{d}-W_{dh}s_{h}\right)}^{2}, \end{array}$$ (20)

or more compactly

$$\begin{array}{cc} n_{d}\left(s_{h}\right) & =\{\begin{array}{cc} l_{d}\left(s_{h}\right) & \text{if}\;s_{h}<P_{d}\\ r_{d}\left(s_{h}\right) & \text{if}\;s_{h}\geq P_{d}, \end{array} \end{array}$$ (21)

which from now on will be referred to as an individual function segment of the entire likelihood function.

Now we generalize the likelihood expression in Eqs (15)–(17) to consider all observed *D* dimensions in **y**. We take the logarithm of $\prod_{d=1}^{D}p(y_{d}|s_{h},{\text{s}}_{H\setminus h},\theta )$, which results in *D*+1 left-piece constant and right-piece quadratic functions to be summed. The sum of all of these pieces will result in the desired *D*-dimensional likelihood function, which will be another piecewise function with *D*+1 disjoint segments. In order to implement the summation of all of these segments efficiently, we need to first sort them by their transition points *P*_*d*_, from smallest to largest values, which we denote by *δ* = *argsort*_*d*_(*P*_*d*_). With this notation, the summation of the pieces of the likelihood can be expressed:

$$\overset{D}{\underset{d}{\sum }}\log p(y_{d}|s_{h},s_{H\backslash h},\theta )$$ (22)

$$\begin{array}{c} =m(s_{h}) \end{array}$$ (23)

$$\begin{array}{c} =\{\begin{array}{cc} m_{1}\left(s_{h}\right) & s<P_{\delta (1)}\\ m_{2}\left(s_{h}\right) & P_{\delta (1)}\leq s<P_{\delta (2)}\\ m_{3}\left(s_{h}\right) & P_{\delta (2)}\leq s<P_{\delta (3)}\\ \vdots & \;\;\;\;\;\;\vdots \\ m_{D+1}\left(s_{h}\right) & P_{\delta (D)}\leq s. \end{array} \end{array}$$ (24)

Importantly, we observe from Eqs (19)–(20) that each segment *m*_*d*_(*s*_*h*_) is a 2nd degree polynomial, which can be represented by computing three coefficients. Thus, we can elegantly compute the operation in Eq (22) as the summation of the coefficients for each segment *m*_*d*_(*s*_*h*_), and since all pieces *l*_*d*_(*s*_*h*_) and *r*_*d*_(*s*_*h*_) are polynomials of 2nd degree, the result is still a 2nd degree polynomial. So for all *D*+1 components of the likelihood in Eq (15), we can compactly formulate Eq (22) with

$$\begin{array}{c} m_{d}\left(s_{h}\right)=\sum_{j=1}^{d-1}r_{\delta (j)}\left(s_{h}\right)+\sum_{u=d}^{D}l_{\delta (u)}\left(s_{h}\right). \end{array}$$ (25)

$$\begin{array}{c} =\overset{D}{\underset{d^{\prime }=1}{\sum }}n_{d^{\prime }}\left(s_{h}\right)\\ \text{for}\;1\leq d\leq D+1 \end{array}$$ (26)

Now that we have computed the difficult part of the posterior, we incoorporate the *spike-and-slab prior* in two steps. The *Gaussian ‘slab’* of the prior is taken into account by adding its 2nd degree polynomial to all the pieces *m*_*d*_(*s*_*h*_), which also ensures that every piece is a Gaussian. The sparsity, or the ‘spike’, will be included only after constructing the full piecewise cumulative distribution function (CDF).

To construct the piecewise CDF, we relate each segment in *m*_*d*_(*s*_*h*_) to the Gaussian ∝ exp(*m*_*d*_(*s*_*h*_)) it defines. Next, the *Bernoulli ‘spike’* of the prior is accounted for by introducing a step into the CDF that corresponds to *s*_*h*_ = 0 (see Fig 3F), where the height of the step is proportional to the marginal probability *p*(*s*_*h*_ = 0∣**s**_∖*h*_). Once the CDF is constructed, we simulate each *s*_*h*_ from the exact conditional distribution (*s*_*h*_ ∼ *p*(*s*_*h*_∣**s**_∖*h*_ = **s**_∖*h*_,**y**, *θ*)) by inverse transform sampling. Fig 3 illustrates the entire process.

**Preselection.** To dramatically improve computational efficiency of inference in our model, we can optionally preselect the most relevant latent variables before doing Gibbs sampling. This can be formulated as a variational approximation to exact inference [16] where the posterior distribution *p*(**s**∣**y** ^(*n*)^, Θ) is approximated by a truncated distribution *q*_*n*_(**s**;Θ) which only has support on a subset 𝓚_*n*_ of the latent state space:

$$\begin{array}{c} p\left(s\;|\;y^{\;(n)},\Theta \right)\approx q_{n}\left(s;\Theta \right)=\frac{p(s\;|\;y^{\;(n)},\Theta )}{\;\int_{s\;{}^{\prime }\in {𝓚}_{n}}\;p\left(s\;{}^{\prime }\;|\;y^{\;(n)},\Theta \right)}\;\delta \left(s\in {𝓚}_{n}\right) \end{array}$$ (27)

where *δ*(**s** ∈ 𝓚_*n*_) = 1 if **s** ∈ 𝓚_*n*_ and zero otherwise. The subsets 𝓚_*n*_ are chosen in a data-driven way using a deterministic *selection function*, they vary per data point **y** ^(*n*)^, and should contain most of the probability mass *p*(**s**∣**y**) while also being significantly smaller than the entire latent space. Using such subsets 𝓚_*n*_, Eq 27 results in good approximations to the posteriors. We define 𝓚_*n*_ as 𝓚_*n*_ = {**s** ∣ for all *h* ∉ ℐ: *s*_*h*_ = 0} where ℐ contains the indices of the latents estimated to be most relevant for **y** ^(*n*)^. To obtain these latent indices we use the cosine similarity as our selection function:

$$\begin{array}{ccc} {𝓢}_{h}\left(y^{\;(n)}\right) & = & \frac{W_{h}^{T}\;y^{\;(n)}}{{\left||W_{h}|\right|}_{2}\;} \end{array}$$ (28)

to select the *H*′ < *H* highest scoring latents for ℐ. This boils down to selecting the *H*′ dictionary elements that are most similar to each data point, hence being most likely to have generated the data point. We then sample from this reduced set of latent variables.

## Results

The above described procedure to optimize the parameters of the nonlinear spike-and-slab model will be referred to as SSMCA. All numerical experiments for SSMCA used a parallel implementation of the EM algorithm for parameter optimization [38], in which for the E-step we use our developed approximate inference scheme based on Gibbs sampling and latent variable preselection. For all described results, 1/3 of the samples are used for burn-in and 2/3 are used for computing the expectations. We initialized our parameters by setting the *σ*_pr_ and *σ* equal to the standard deviation observed in the data, the prior mean *μ*_pr_ is initialized to the observed data mean. **W** is initialized at the observed data mean with additive Gaussian noise of the *σ* observed in the data.

### Parameter recovery on artificial ground-truth data

**Fig 4:**
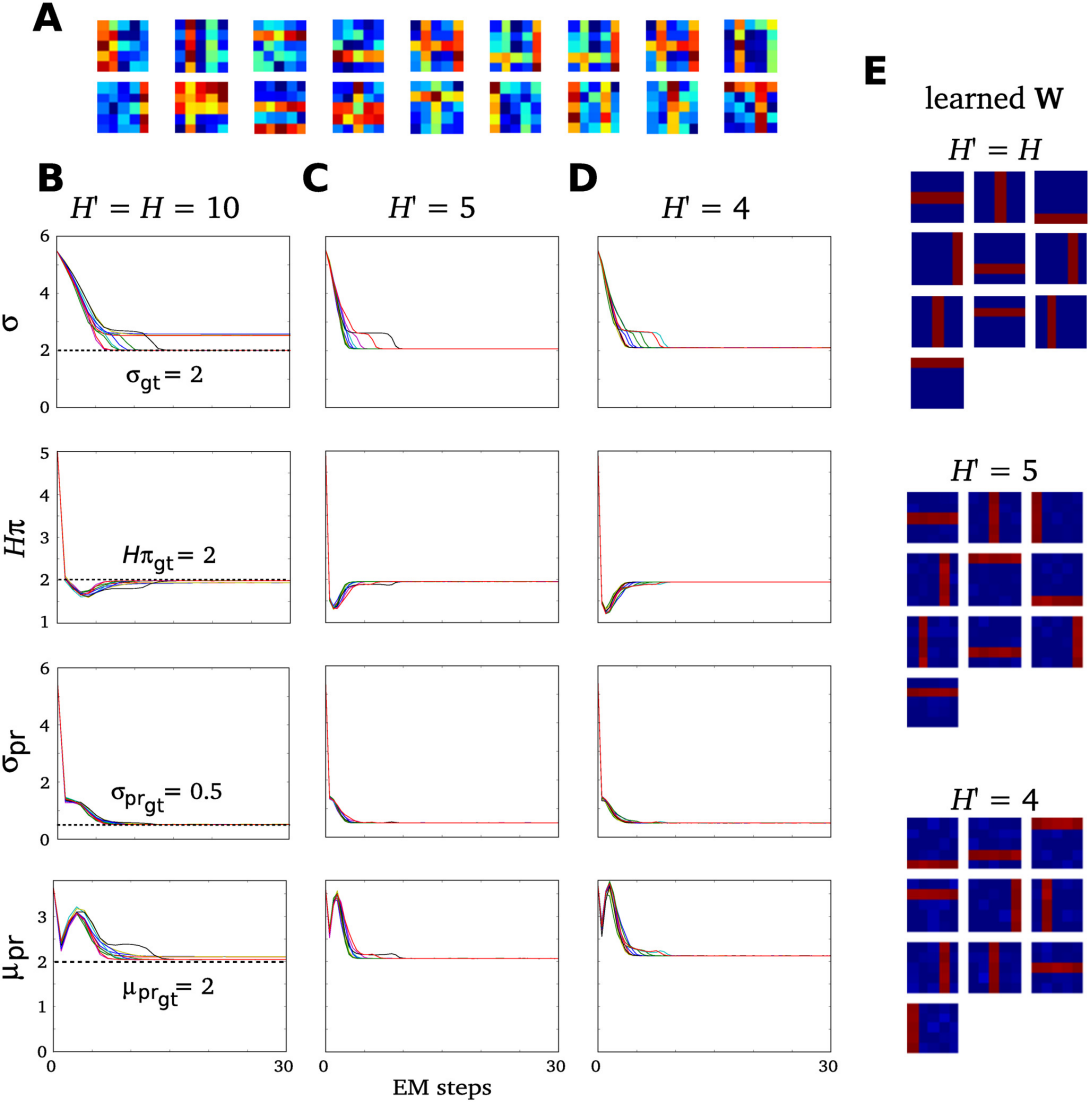
Parameter recovery on synthetic data. Results of three differently parameterized sets of experiments, each with 10 experimental runs of 30 EM iterations on identical artificial ground-truth data generated according to the SSMCA model: **A** *N* = 2,000, *D* = 5 × 5. Three shown experimental settings are: **B** *H*′ = *H* = 10, **C** *H*′ = 5, and **D** *H*′ = 4, although the same results were obtained by the entire range of parameters *H*′ = [4, 10]. Importantly, the figure shows accurate recovery of ground-truth parameters which are plotted with dotted lines. **B**, **C** and **D** show in each column the parameter convergence of each of the three experiments, where the rows contain the following: data noise *σ*, sparsity *H* × *π*, prior standard dev. *σ*_pr_, and the prior mean *μ*_pr_. Finally, **E** shows the set of learned generative fields/components **W**_*h*_ corresponding to each experimental set **B** *H*′ = *H* = 10, **C** *H*′ = 5, and **D** *H*′ = 4.

The goal of the first set of experiments is to verify that our model and inference method produce an algorithm that can Eq (1) recover ground-truth parameters Θ = (*π*, *μ*_pr_, *σ*_pr_, *W*, *σ*) from data that is generated according to the model and Eq (2) that it reliably converges to locally (if not globally) optimal solutions. We generate ground-truth data with *N* = 2,000 consisting of *D* = 5 × 5 = 25 observed and *H* = 10 hidden dimensions according to our model: *N* images with overlapping ‘bars’ of varying intensities and with Gaussian observation noise of variance *σ*_*gt*_ = 2 (Fig 4A). On average, each data point contains two bars, $\pi =\frac{2}{H}$.

First, we optimize the model using just Gibbs sampling, which aims to do inference as exactly as possible in this model. Namely, we do sampling without preselection and draw samples from the entire latent space: we set the preselection parameter *H*′ = *H* and draw 30 samples from the full *H*-dimensional posterior. After this, we evaluate our combined approximate inference approach of preselection and Gibbs sampling. Results (Fig 4B and 4E) show that our algorithm converges quickly and recovers the generating ground-truth parameters.

Next, we investigate a range of numbers of samples drawn and consider the range of preselected latent variables *H*′ ∈ (4,10) from the entire *H*-dimensional posterior space. These experiments yield the same results: our algorithm reliably converges quickly to (at least) locally optimal solutions of all parameters in all runs of the experiments with 30 EM iterations. This suggests that our approximation parameters do not strongly affect the accuracy of our inference results. See Fig (Fig 4C, 4D, 4E) for some further convergence examples, namely where *H*′ = 4 and *H*′ = 5.

### Occlusions data: Dictionary learning and image reconstruction

In order to directly evaluate the differences between our nonlinear SSMCA model and the standard linear sparse coding model (which will be referred to as LinSC), we consider dictionary learning and image reconstruction on two datasets consisting of true occlusions. Here the task is to learn the set of components **W**, i.e. the dictionary elements, that are behind the composition of a given observed dataset **y**, and consider reconstruction of individual images/data points **y** ^(*n*)^. The goal of these experiments is to understand how the learned components are affected by the models’ assumptions and furthermore the effect this has on the quality of the image reconstruction.

For the linear SC comparison we use the sparse online dictionary learning algorithm [39], which is a state-of-the-art matrix factorization sparse coding approach and is based on the objective function formulated in Eq (1). Furthermore, in order to study the effect of the spike-and-slab prior, we apply the SSMCA algorithm with a narrow and fixed prior slab (small variance for the Gaussian of the prior distribution). Such a fixed narrow slab approximates a binary prior. Binary priors have thus far been used with nonlinear approaches ([16, 19, 20, 25, 40]) including previous MCA versions [16, 19, 20]. We will refer to the SSMCA algorithm with fixed narrow slab as SSMCA^fix^. To make sure that the differences in the results using SSMCA^fix^ vs. SSMCA can be attributed to the difference between binary-like and spike-and-slab prior, we make sure that SSMCA and SSMCA^fix^ are identical except for the algorithmic aspects concerned with learning the slab. Note that the data model underlying SSMCA^fix^ connects to that of standard MCA [16, 20] and becomes identical in the limit of an infinitely narrow slab (a delta peak). However, the algorithms for parameter optimization remain different also in this limit (SSMCA^fix^ remains sampling based, for instance).

#### Realistic occlusion dataset

**Fig 5:**
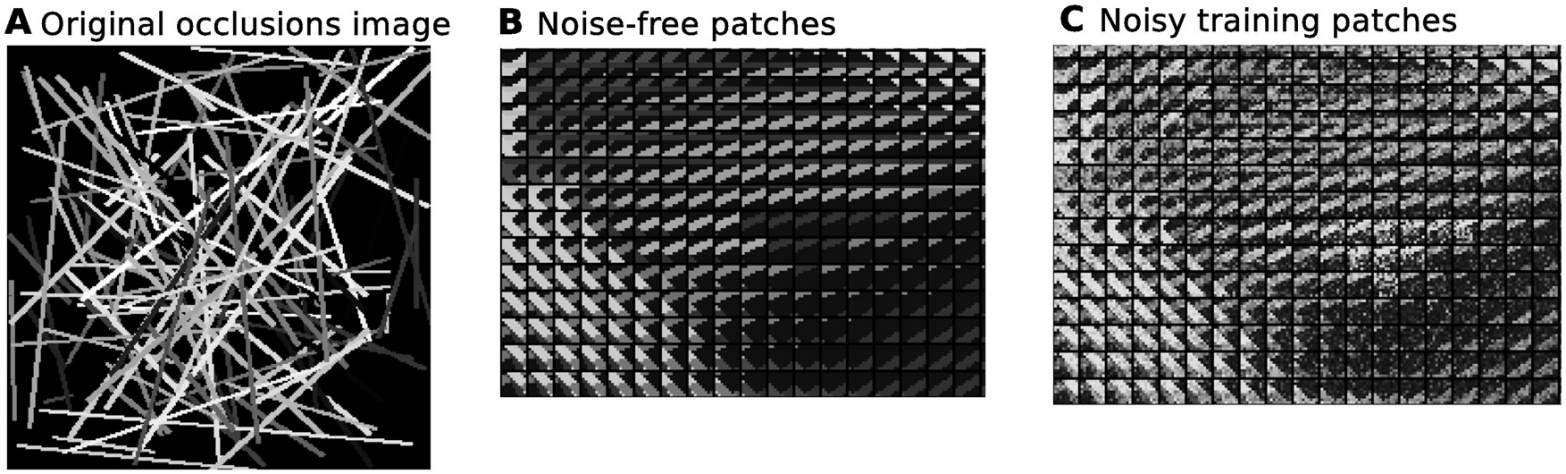
Synthetic occlusion dataset and cut–out original and noisy patches. Examples taken from the occlusion dataset. **A** shows an original noise-free image of generated occluding strokes of random width, pixel intensity, and starting/ending points. **B** shows a handfull of overlapping image patches cut from the original, noise-free data. **C** shows examples of the noisy training data, with independent *σ* = 25 noise added to **B**.

The first dataset we compare the algorithms on is one with controlled forms of sparse structure, a realistic artificial dataset of true occlusions (data created by actual occlusions and not following any model considered here). The data was generated using the Python Image Library (PIL) to draw hundreds of overlapping edges/strokes in a 256 × 256 pixel image: each stroke had an integer intensity between (1,255), a width between (2,4) pixels, and a length, starting, and ending position drawn independently from a uniform distribution. The image was then cut into overlapping *D* = 9 × 9 patches, each of which contained *k* ∈ (0,5) overlapping strokes, for *N* = 61009. Gaussian observation noise of *σ* = 25 and *μ* = 0 was then independently added to each patch, thus concluding the considered occlusion dataset. Examples are shown in Fig 5. Additionally, the dataset also contains the corresponding (automatically obtained) labels for each image, indicating the *ground-truth number of occluding strokes* *k* ∈ (0,5) per image.

Such a dataset represents and isolates challenging aspects of low-level image statistics that are present in all natural images. Particularly, it contains edges of varying intensities and their occlusions. We have selected it because it is complex enough to narrow in on the consequences of the different model assumptions, but simple enough that we know what generated/caused the data. In this way, we can interpret the results and evaluate what each approach learns, particularly how they cope with occlusions.

We run the nonlinear SSMCA and the linear SC methods on the occlusions data set. We set the number of dictionary elements to be learned from the dataset to *H* = 100, but we also ran experiments learning larger (*H* = 256) dictionaries, which yielded the same results for both the linear and nonlinear methods. For SSMCA and SSMCA^fix^ we draw 40 samples per data point, per variable (i.e. 40 × 100 = 40000 samples per data point when sampling 100 variables). The number of preselected latent variables was set to *H*′ = 10 with 2 randomly chosen variables each iteration. For LinSC, we used regularization parameters *a* = (1,50,100) in Eq 1 in order to evaluate the reconstruction and the components learned across a range of sparse solutions.

**Fig 6:**
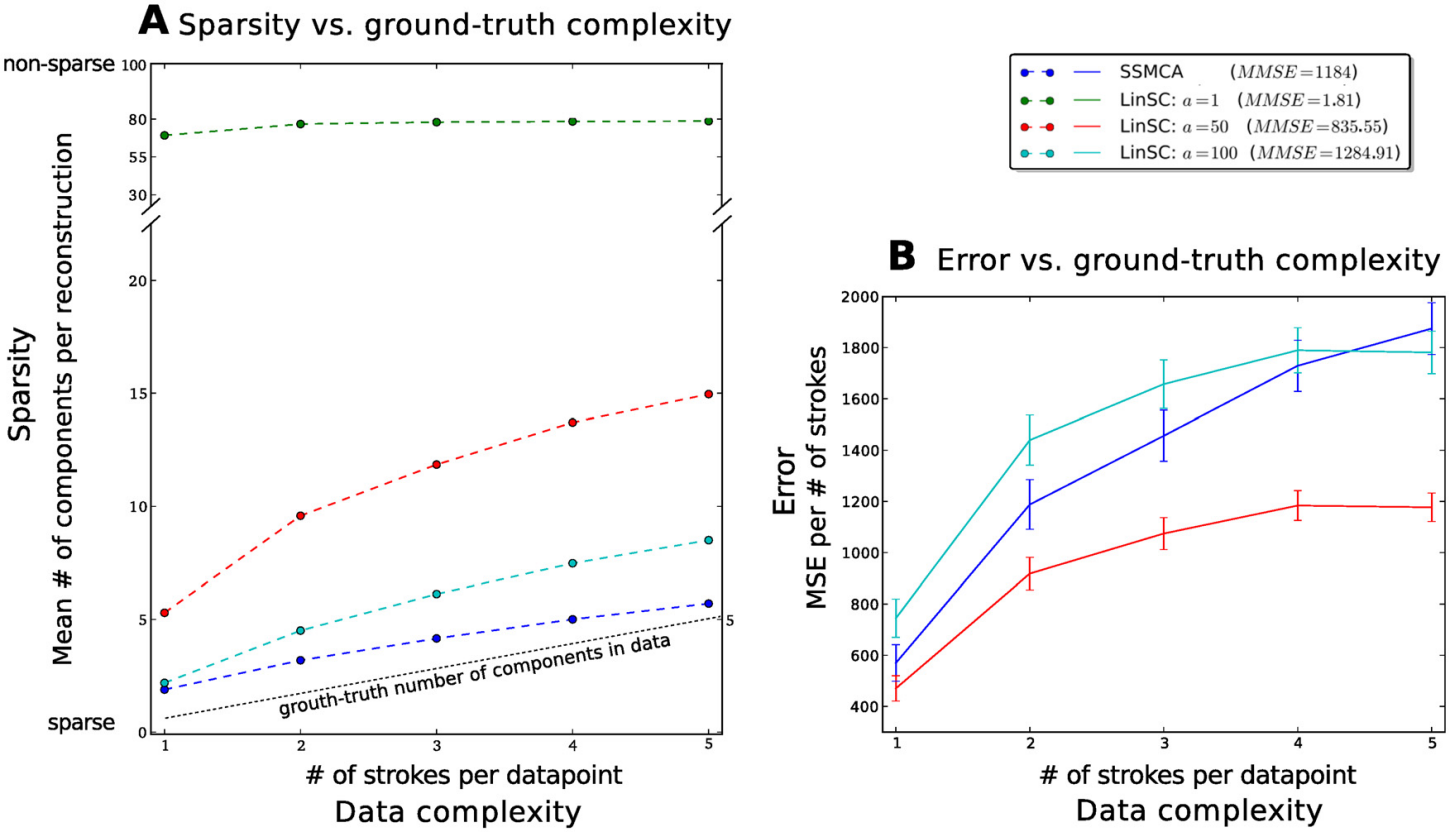
Comparative experiments of linear and nonlinear sparse coding on dictionary learning and image reconstruction. With *H* = 100 learned dictionary components we evaluate the number learned and used for reconstruction. **A** shows the relationship between sparsity (number of components used for reconstruction) and data complexity (number of strokes in the data). Interestingly, the SSMCA plot (blue curves) shows a nearly linear relationship of the number of components used for reconstruction versus the number of components (strokes) actually in the data, suggesting that reconstruction-complexity of the data by nonlinear model more closely follows the actual complexity in the data. On the contrary, the linear parameterization that yields good reconstruction results *a* = 1 shown in green, does not adapt to the data complexity at all: it consistently uses nearly 80 of the learned 100 components per reconstruction, regardless of the data point’s actual complexity (note the change in scale of the *y*-axis around 30 components in order to fit the green curve on the plot). **B** shows the relationship of the mean squared error (MSE) of the reconstructions of all versus the corresponding data complexity (number of strokes in the data). When the reconstruction-complexity (sparsity) is far from the actual complexity of the data (linear methods: red, *a* = 50 and green *a* = 1 cases) the MSE improves. However, when the sparsity is more closely matched to the data, SSMCA and the weakly regularized linear methods result in a poorer MSE. SSMCA nevertheless yields a better MSE in this case, even when it and linSC *a* = 100 have a very similarly sparse solutions/use the same number of components. Note that the error of the least sparse LinSC approach (*a* = 1) is so low (mean MSE = 1.81), it does not even appear on this graph. Error bars shown are scaled to be 10% of the standard deviation for all methods in all stroke-complexity cases. The mean MSE (averaged over the entire dataset) is shown in the legend next to the respective algorithm.

The results showcase a number of notable effects. First, we see in Fig 6A the relationship between sparsity (number of components used for reconstruction) and data complexity (*k* number of strokes in the data). The complexity of the data reconstruction by SSMCA more closely follows the actual complexity in the data: the SSMCA plot (blue curves) shows a nearly linear relationship of the number of components used for reconstruction versus the number of components (strokes) actually in the data. In other words, although all methods adapt the number of fields used for reconstruction to the complexity of the data, our approach adapts to the extent of using nearly only as many components as are actually in the image (according to ground-truth). Furthermore, Fig 6B shows the relationship of the reconstruction quality versus the corresponding data complexity, in terms of the *k* number of strokes in the data. We quantify the quality of reconstruction with the mean squared error (MSE, $\sum_{n}{\left({\text{x}}_{n}-{\hat{\text{x}}}_{n}\right)}^{2}$, or the mean MSE, MMSE, which is MSE averaged over the respective dataset), which is very sensitive to subtle variances in an image versus its reconstruction. Notably, when the linear method is regularized to yield a solution as sparse as the nonlinear method (LinSC *a* = 100, cyan curves), its reconstruction MSE suffers.

**Fig 7:**
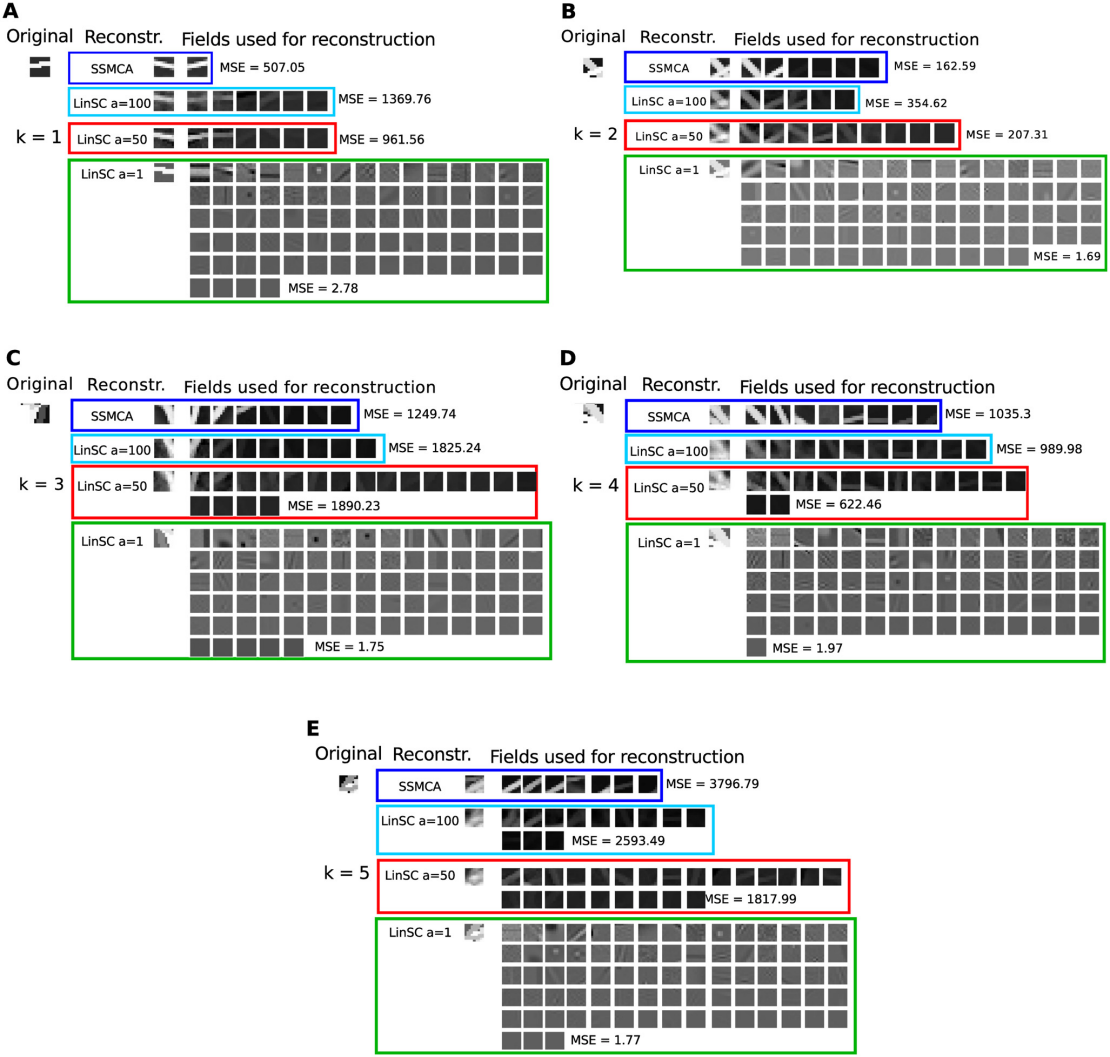
Comparison of linear and nonlinear sparse coding on image reconstruction. Shown are a handfull of real data points of varying complexity in terms of the number of strokes *k* in each image (*k* ∈ (1,5) strokes per image), the components/fields learned by the various algorithms, the corresponding reconstruction of the given data point, and the mean squared error (MSE) of each reconstruction. **A** image with *k* = 1 stroke, **B** *k* = 2 strokes, **C** *k* = 3 strokes, **D** *k* = 4 strokes, and **E** *k* = 5 strokes. Regardless of image complexity—how many causes/strokes are in an image—the components used by the nonlinear method (SSMCA) resemble the true causes of the image: each component contains a single, interpretable stroke. On the other hand, none of the *a* parameterizations of the linear method yield stroke-like components, even when the solution is regularized to be as sparse as SSMCA (*a* = 100). Note: all images in the *a* = 1 case appear brighter than they actually are, due to visualization with a python toolbox, but are in reality of the identical brightness scale to the original data point (and all other shown cases), hence the reconstruction error (MSE) is very low.

Next, we investigate the actual components each model uses in order to reconstruct a given image patch. Fig 7A–7E contains a comparison of the reconstruction of a handful of image patches by the linear and the nonlinear methods. Evaluation of the fields/components learned by each method suggests that the nonlinear max, which aims to model occlusions, is better able to learn generating causes of the occlusion-rich images. Regardless of image complexity—how many causes/strokes are in an image—the components used by the nonlinear method (SSMCA) resemble the true causes of the image: each component contains a single, interpretable stroke. On the other hand, none of the *a* parameterizations of the linear method yield stroke-like components, even when the solution is regularized to be as sparse as SSMCA. For example, if we just consider sparse solutions, namely compare the methods which use fewest components for reconstruction (SSMCA and LinSC with *a* = 100; blue and cyan curves, respectively), we see that not only is the nonlinear SSMCA solution consistently better in terms of MSE, but also the components learned/used are very different.

**Fig 8:**
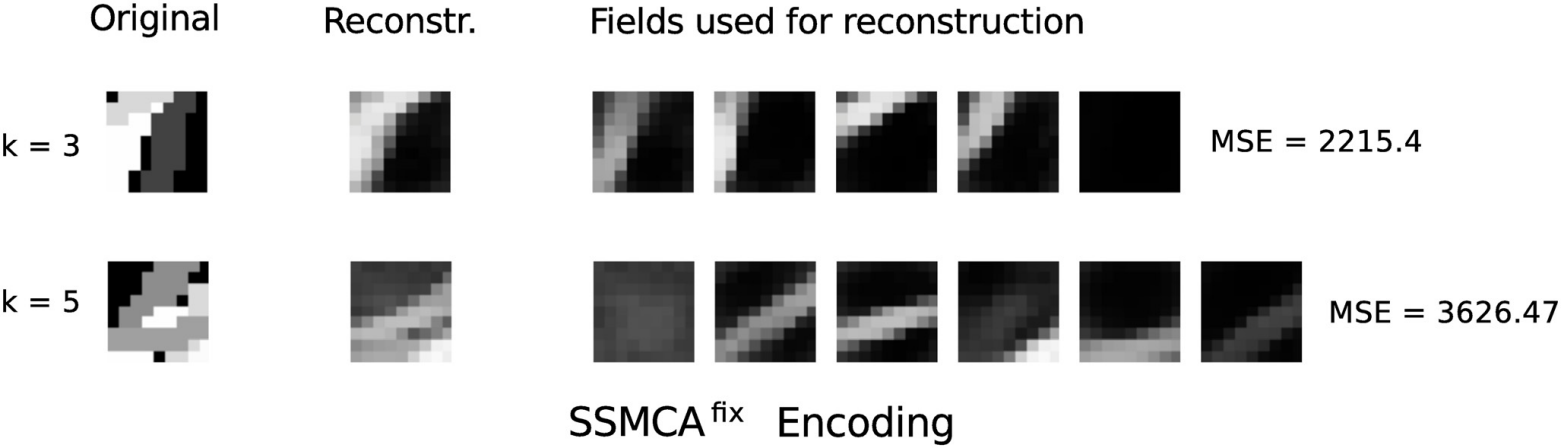
Results of nonlinear sparse coding using a binary prior on image reconstruction. The nonlinear sparse coding model if applied to artificial strokes using a fixed narrow slab (SSMCA^fix^. The two figure columns show image reconstruction results for SSMCA^fix^ with *σ*_pr_ = 0.25 for two different ground-truth stroke numbers (*k* = 3 and *k* = 5). SSMCA^fix^ was first trained with fixed *σ*_pr_ and then applied to the data. Reconstructions were computed as described for Fig 7.

Although SSMCA extracts components resembling the generating causes, in some cases the reconstruction MSE suffers because the model does not allow for error correction via adding negative components (which, if it did allow for such corrections, would furthermore lead to a less sparse solution). In contrast, the linear methods are optimized for the best image reconstruction MSE using summation (as can be seen in the method’s objective function in Eq (1)), and consequently are able to learn a set of components which can be added/subtracted for the optimal MSE. This is particularly evident in the linear *a* = 1 case (green plots/highlighting), where sparsity is weakly enforced, and thus a larger set of components can be used to fine-tune a near-perfect reconstruction of the original image. Components learned by a control run with SSMCA^fix^ with *σ*_pr_ fixed to 0.25 look similar to those learned by SSMCA and quite different to the ones of linear sparse coding (see Fig 8 for some examples). The learned sparsity is also similar to the one inferred by SSMCA but we observed only weak scaling with the complexity of the patches (for *σ*_pr_ ≥ 0.25) to no scaling (for *σ*_pr_ ≤ 0.25). Also the sparsity values were consistently higher for SSMCA^fix^ compared to SSMCA (i.e., fewer components for SSMCA^fix^). Furthermore, the average image reconstruction errors significantly increases for SSMCA^fix^ compared to SSMCA (e.g. for *σ*_pr_ = 0.25 we get a MMSE of 1833). The significant increase in reconstruction error is due to a decreased ability to fine-tune dictionary coefficients to the intensities of the components—the intensity range from which the coefficients can assume is lower. For the SSMCA^fix^ algorithm this also seems to indirectly influence the learned sparsity, maybe due to SSMCA^fix^ attributing components with a low pixel intensity to background noise. We observe the reconstruction errors and sparsity to increase when we decrease the width of the fixed slab (namely, when decreasing *σ*_pr_).

Regarding all the results reported here, note that the max is also just an approximation of the true occlusion combination rule. If a dark stroke is occluding a brighter stroke, for instance, the true gray-value of the overlapping region is not reproduced by the max. Still, the SSMCA reconstruction is (given ground-truth strokes as dictionary elements) at least as good as in the linear case, and better except for boundary cases. Therefore, it seems to be easier for the nonlinear model to learn dictionary elements close to the generating components, i.e. interpretable components.

To summarize, SSMCA extracts meaningful, interpretable components—components closely match the generating process, adapts to complexity in the data, as measured by the number of strokes/edge components in an image, and uses correspondingly more or fewer components for the reconstruction. The reconstruction solution SSMCA offers is much sparser than that of LinSC, for any levels of reconstruction error (MSE).

As a control, we also ran the same set of experiments, but varying *H* and *H*′—learning a larger set of latent components (dictionary set) *H* and ranging the SSMCA preselection parameter *H*′ values—all of which resulted in the same trends shown in Figs 6 and 7.

#### Natural image occlusions

**Fig 9:**
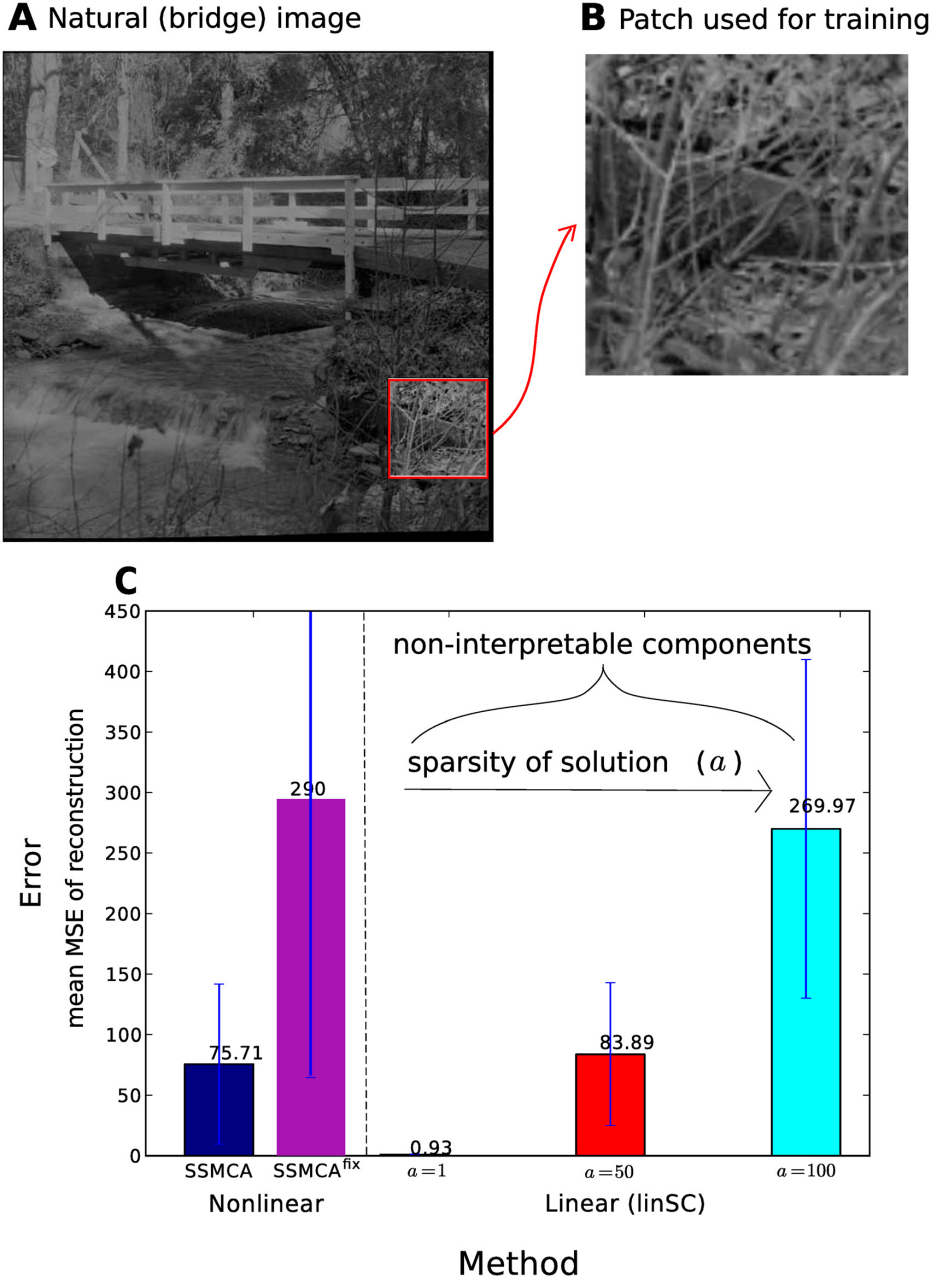
Results of comparative experiments of linear and nonlinear sparse coding methods on component learning/image reconstruction on natural image patches. **A** shows the original natural image data, bridge.jpg [39], from which we cut an occlusion-rich underbrush region. **B** shows the original section taken from **A**, scaled up to 256 × 256 pixels, which was then cut into overlapping patches and given independent Gaussian noise with *σ* = 5 to compose the considered dataset. **C** shows the mean squared error (MSE) of the compared nonlinear and linear methods’ reconstruction averaged over the entire dataset, with the standard deviation indicated with error bars. The trend is the same as in the artificial occlusions data experiments: the nonlinear method maintains reasonably low MSE, while learning a sparse set of interpretable components, whereas the linear method achieves a very low MSE only when it does not learn a sparse (and never interpretable) solution of components.

We have shown that our approach can model realistic artificial occlusions well. Now we are interested in investigating the performance of the linear and nonlinear approaches on naturally occurring occlusions. We use an image of underbrush in a forest (taken bridge.jpg, which has been used for denoising benchmarking [39]), which is rich with occluding branches and twigs. See Fig 9A for the original noise-free image, from which we cut a 110 × 110 pixel occlusion-rich section and scaled it up to 256 × 256 pixels to use in our dataset, shown in Fig 9B. To compose the dataset as in the previous experiments, we cut the 256 × 256 image, with pixel values *x*_*i*_ ranging from (0,255), into *N* = 61009 overlapping image patches of *D* = 9 × 9 pixels, then add independent Gaussian noise with *σ* = 5. We run the exact same set of experiments as with the original occlusions dataset, with both the nonlinear and linear methods learning a dictionary size of *H* = 100 latents. For SSMCA we again draw 40 samples per data point, per variable (i.e. 40 × 100 samples per data point), and set the number of preselected latent variables to *H*′ = 10 with 2 randomly chosen per iteration. For linear SC, we again used regularization parameters *a* = (1,50,100).

Because we do not have any ground-truth associated with this dataset as to how many strokes/components are in a given image, we can only compare the average reconstruction error for the entire dataset across methods. Fig 9C shows the mean MSE of each method with the associated standard deviation. The results follow the trend outlined in the previous set of experiments (in Fig 6B), where again if LinSC uses as sparse a reconstruction as SSMCA (in *a* = 100 case), the mean reconstruction error is far poorer than that of SSMCA (MMSE = 269.96 vs. MMSE = 75.71). Furthermore, even when LinSC is less sparse (in *a* = 50 case), the mean reconstruction error is still slightly poorer than SSMCA (MMSE = 83.89 vs. MMSE = 75.71). On the other hand, when the linear model uses a highly non-sparse solution (LinSC *a* = 50, resulting in using 75 of 100 components for reconstruction), it can fine-tune its reconstruction to achieve very low error (MMSE = 0.93). However, the components each linear model uses for reconstruction are non-interpretable (e.g. do not resemble edge-like structures) for any of the linear models, regardless of their sparsity or reconstruction error both nonlinear models use components that indeed resemble edge-like structures and are interpretable.

When applying SSMCA^fix^ as a control, the learned dictionary components are similar to the ones by SSMCA, however, the reconstruction error is much worse than that of SSMCA with an MMSE of 290 for *σ*_pr_ = 0.25 (see Fig 6 for comparison). When we make the slab still narrower, the reconstruction error further increases (e.g., MMSE = 377 for *σ*_pr_ = 0.1), which is consistent with a reduction of the ability to accurately match the varying stroke intensities using continuous coefficients.

### Natural image patches and neural consistency

Understanding the encoding provided by sparse coding and its capability to extract interpretable data components is important for functional applications but, furthermore, also of high relevance for probabilistic models of the primary visual cortex (V1). Since the seminal study by [41] sparse coding can be considered as a standard model for the response properties of V1 simple cells. Evidence that response properties of V1 simple cells may be better described by a sparse coding model that reflects occlusions has been provided by a recent comparative study [19]. To complete our investigation of the SSMCA model, we will apply it to the same data as used in that study. In contrast to the binary sources assumed by [19], our model allows us to study the statistics of basis functions under the standard assumption of continuous latents.

We apply our model to *N* = 50,000 image patches of *D* = 16 × 16 = 256 pixels and learn *H* = 500 hidden dimensions/generative fields, and run 50 EM iterations with 100 samples per data point. The patches were extracted from the van Hateren natural image database [42] and subsequently preprocessed using pseudo-whitening [4]. We split the image patches into a positive and negative channel to ensure *y*_*d*_ ≥ 0: each image patch $\tilde{y}$ of size $\tilde{D}=16\times 16$ is converted into a data point of size $D=2\;\tilde{D}$ by assigning $y_{d}={\left[{\tilde{y}}_{d}\right]}^{+}$ and $y_{\tilde{D}+d}={\left[-{\tilde{y}}_{d}\right]}^{+}$, where [*x*]^+^ = *x* for *x* > 0 and [*x*]^+^ = 0 otherwise. This can be motivated by the transfer of visual information by center-on and center-off cells of the mammalian lateral geniculate nucleus (LGN). In a final step, as a form of local contrast normalization, we scaled each image patch so that 0 ≤ *y*_*d*_ ≤ 10.

**Fig 10:**
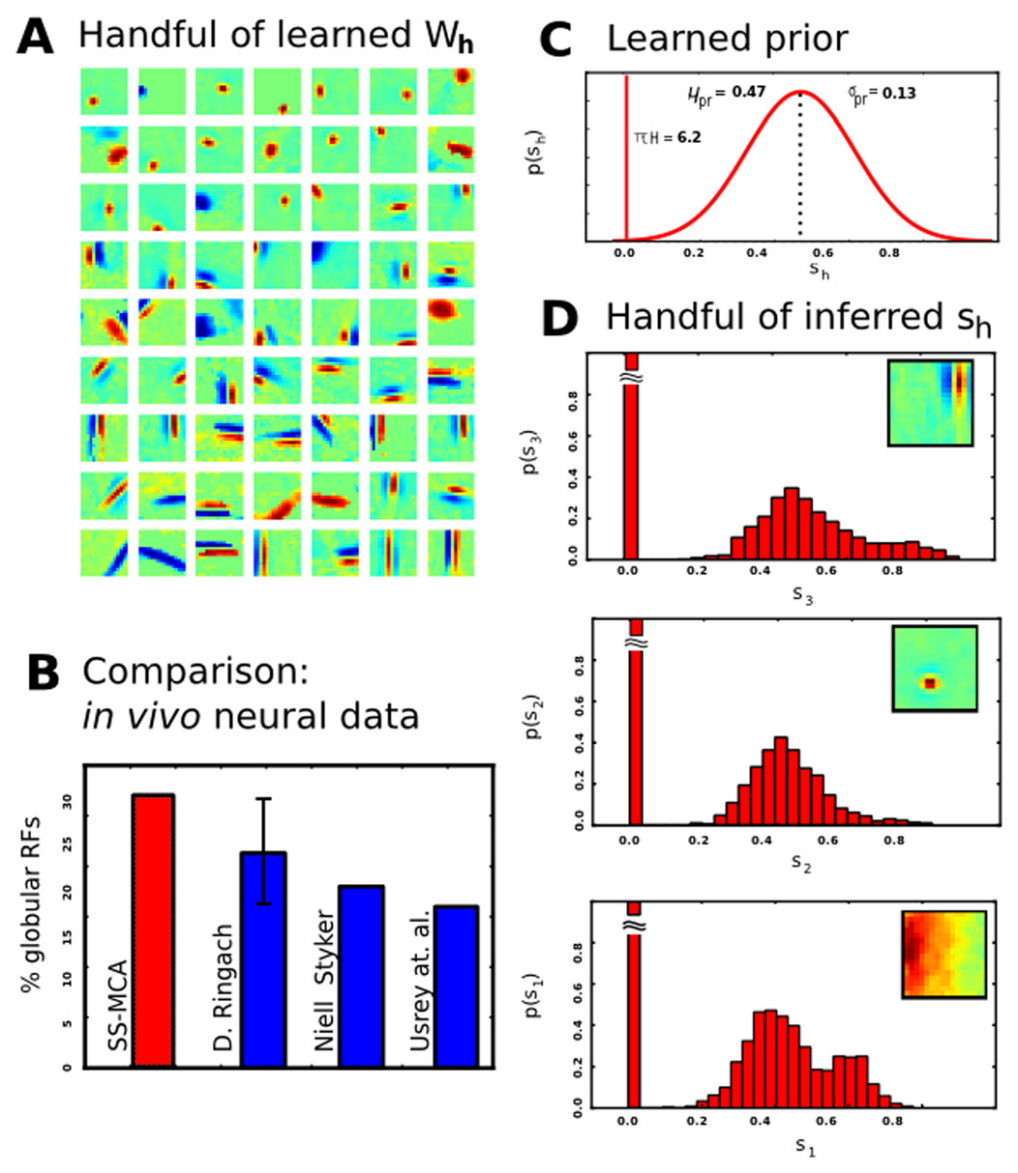
Analysis of dictionary components learned by the SSMCA algorithm on natural image patches. **A** Example dictionary elements **W**_*h*_ after learning. **B** Fraction of globular fields estimated from *in vivo* measurements, compared to ours (after fitting with Gabor wavelets and DoG’s; globular percentages taken from [19] who analyzed data provided by [18] and estimated percentages of globular fields from data in two further papers [43, 44]. **C** Learned prior. **D** Actual activations of diverse dictionary elements *s*_*h*_ (posterior averaged over data points).

All results are shown in Fig 10. In Fig 10A, we have a handful of the learned dictionary elements **W**_*h*_ (which are a variety of Gabor-Wavelet and Difference of Gaussians (DoG)-like shapes). To quantitatively interpret the learned fields, we perform reverse correlation on the learned generative fields and fit the resulting estimated receptive fields with Gabor wavelets and DoGs (see S1 Results 2 for details). Next, we classify the fields as either orientation-sensitive Gabor wavelets or ‘globular’ fields best matched by DoGs. In Fig 10B we compare the percentages of ‘globular’ fields to *in vivo* recordings. These results are consistent with neural recordings: notably, the proportion of DoG-like fields in the same high range as the proportions found in different species [18, 43, 44] (See [19] for data and a discussion), which is a result not observed by the established linear SC variants. The learned prior and its parameters are shown in Fig 10C: learned sparseness was *πH* = 6.2 (i.e. on average six active latent variables per image patch), mean *μ*_*pr*_ = 0.47, with standard deviation *σ*_*pr*_ = 0.13. The learned data noise was *σ* = 1.4. Exhibiting consistency with the learned prior, Fig 10D shows a handfull of the inferred latent variables (coefficients) *s*_*h*_. These correspond to the actual activations of the diverse dictionary elements **W**_*h*_, each of which is visualized in the upper right of each subfigure. A part of these results have also appeared in a preliminary application of this model in a conference submission [22]. Please see S1 Results 2 for the complete set of generative fields learned and for a larger set of the learned prior activations.

Since we have shown consistent predictions with neural recordings, we finally test the model for consistency with the natural image patches dataset. Specifically, we are interested in consistency of the prior beliefs with inferred beliefs, as it is a necessary condition of the correct data model that the posterior averaged over the data points **y** ^(*n*)^ matches the prior (compare e.g. [45]):

$$\underset{N\to \infty }{\lim}\begin{array}{c} \frac{1}{N}\sum_{n}p(s|y^{\left(n\right)},\Theta )=p(s|\Theta ). \end{array}$$ (29)

After the learning on image patches as described above, we observed that averaged posteriors over data points closely resemble the learned prior (see Fig 10E for examples). Linear sparse coding has reportedly struggled with this consistency condition (see [36] for a discussion).

## Discussion

In this work we introduced a sparse coding model that modifies standard sparse coding in two ways: it uses a spike-and-slab distribution instead of a Laplace prior and the nonlinear max superposition instead of the standard linear superposition. With these additions, the proposed model can realistically model low-level image effects. Particularly, the nonlinearity of the max equips the approach to well-approximate occlusions.

As learning and inference in a model with these two modifications is difficult, we also proposed a combined preselection-sampling scheme that constructs the conditional posterior with high accuracy and efficiency. This inference approach allowed us to apply, for the first time, a sparse coding model with continuous latents and strongly nonlinear combination to reasonably high-dimensional observed and hidden space dimensions. The approach is therefore applicable to the typical application domains of standard sparse coding. Furthermore, it offers itself as a novel model for neural responses that encode component intensities. Unlike (linear and nonlinear) models with binary latents [19, 46, 47], it can capture a more fine-tuned representation of sensory stimuli.

Our main interest in this work was in gaining deeper understanding of the consequences of the component combination assumption (linear or nonlinear) and to highlight these consequences empirically in numerical experiments. First, in experiments on artificial data, we have shown that the model and inference approach can learn ground truth parameters. Furthermore, using experiments on natural image patches, we showed consistency of our model in two ways: its predictions are consistent with Eq (1) *in vivo* neural recordings and with Eq (2) its prior beliefs. Our experiments on dictionary learning and image reconstruction show, as the crucial difference, that the nonlinear method learns and uses interpretable image components when reconstructing a given image patch (unlike the linear method [39]). Namely, we have defined ‘*interpretable*’ to mean that the extracted components *closely match the generating process*. Furthermore, we have shown that our method adapts to complexity in the data and uses correspondingly more or fewer components for the reconstruction. Not only does our method yield meaningful and adaptive solutions, but its solution is always much sparser than that of any of the comparable parameterizations of the linear SC method, for any levels of corresponding reconstruction error (MSE).

Our results consequently show that the max nonlinearity is sufficient to reproduce many properties desired from a hidden causes approach to image patch modeling—especially “interpretable” encoding. Future work could go even further, e.g., by taking into account object depths for a more explicit occlusion modeling. The challenges for inference are significantly increasing in this case as unconstrained object permutations result in a super-exponential scaling of hidden states. While explicit occlusion models can be developed based on similar methods as used here (see [48] and citations therein), a combination with a prior for continuous variables such as the spike-and-slab distribution, poses a considerable and yet to be mastered scientific challenge. Work using the max nonlinearity and related approaches, therefore, focuses on capturing the essential properties of occlusion with more compact models, which allow for larger-scale applications comparable, e.g., to those possible with linear sparse coding approaches. Work by Zoran and Weiss [49] aims at capturing occlusion nonlinearities using a mixture model approach, and a comparison with linear models shows significant advantages and improved interpretability. Other recent work combines translation invariance with the exclusiveness property of occlusion [31]. Although that work offers a multiple-causes approach as the one proposed here, they do not provide a model for a continuous distribution of component intensities. Linear approaches are also being continuously further developed. By using a massive increase in the number of hidden units [50], it can be observed that, e.g., globular components (compare with the Results section on Natural image patches) can also emerge using linear approaches (also see [19] for a discussion). In such highly overcomplete settings, sparsity can be increased, which tends to increase the interpretability.

Further linear approaches include non-negative matrix factorization (NMF) methods which are usually not formulated probabilistically. Previous work [20] quantitatively compared different NMF versions to MCA with binary hidden units, which itself is approximated by the SSMCA^fix^ evaluated as a control here. Using the bars benchmark test (which can be considered as a simplified version of the data used in the dictionary learning and image reconstruction experiments, see Results), it was shown that MCA performs well in this nonlinear task. Already for the comparably simple bars test, standard NMF was shown to fail (experimental data from [51]). Only if constrained appropriately, using hand-tuned constraints on sparsity for weights and latent activity, NMF was reported to learn the correct generating components. Such constrained extensions for NMF objective functions can be combined with any noise metric (e.g., Poisson, Gaussian, Manhatten; compare [52, 53]), but the sparsity parameter in these approaches is hand-fixed and cannot be learned. In a probabilistic formulation, constraints could be reformulated as priors and indeed be learned, which could potentially make them more similar to the approach used here. Regarding the inherent superposition assumption, all NMF approaches are, by definition, linear and thus in this respect more similar to linear sparse coding than to SSMCA. Consequently, the linearity assumption used by NMF could explain why, e.g., the algorithm requires additional mechanisms in order to learn the correct solution for the bars experiments.

In conclusion, this work marks first steps in uncovering the benefits and drawbacks of the implicit assumptions made within sparse coding models, the understanding of which will help researchers to select the most suitable model for their task. If the primary goal is for image reconstruction, our experiments suggest the linear model to be the better choice, whereas if the goal is to extract a sparse dictionary set approximating the data generation, our approach would be more beneficial.

## Supporting Information

S1 Derivation M-step Parameter Equation Derivations. The equations computed every EM iteration in the M-step to update the model parameters to the current maximum likelihood solution are shown here with their derivations.
(PDF)

S1 Results Experiments: Natural Image Patches. The complete set of generative fields learned *W* learned in the experiments on natural image patches and a larger set of the learned prior activations are shown here.
(PDF)
