# Supplementary material for: Nonlinear Spike-And-Slab Sparse Coding for Interpretable Image Encoding
Source: PLoS One. 2015 May 8;10(5):e0124088. doi: 10.1371/journal.pone.0124088 (PMC4425358; doi:10.1371/journal.pone.0124088)
Supplement: S1 Derivation — The equations computed every EM iteration in the M-step to update the model parameters to the current maximum likelihood solution are shown here with their derivations. (PDF) [file pone.0124088.s001.pdf]

S1 Derivation

M-Step Parameter Equation Derivations

The optimal parameters that maximize the data log-likelihood under the generative model can be sought by Expectation Maximization (EM) algorithm (see eg., [1]), which iteratively optimizes a lower bound $\mathcal{F}(\Theta, q)$ of the likelihood w.r.t. the parameters Θ and a distribution q:

$$\mathcal{L}(\Theta) \geq \mathcal{F}(\Theta, q_{\Theta'}) = \sum_{n=1}^{N} \sum_{s} q_n(\vec{s}|\Theta') \log \frac{p(y^{(n)}, \vec{s}|\Theta)}{q_n(\vec{s}|\Theta')} \tag{1}$$

$$= \langle \log p(\vec{y}, \vec{s} \mid \Theta) \rangle_{q(\vec{s}|\Theta')} + \mathrm{H}[q(\vec{s}|\Theta')]. \tag{2}$$

Each iteration consists of an E-step and an M-step. The E-step optimizes the lower bound w.r.t. to the distributions $q_n(s \mid \Theta)$ by setting them equal to the posterior distributions $q_n(s \mid \Theta) \leftarrow p(s \mid y^{(n)}, \Theta)$ while keeping the parameters Θ fixed, denoted by Θ'. The M-step then optimizes $\mathcal{F}(\Theta, q_{\Theta'})$ w.r.t. the parameters Θ keeping the distributions $q_n(s \mid \Theta')$ fixed. If we are given many samples of s for the posterior then we wish to find:

$$\Theta^{(t+1)} = \mathrm{argmax}_{\Theta} \mathcal{F}(\Theta, q_{\Theta^{(t)}}). \tag{3}$$

This is maximised with the maximum likelihood estimate:

$$\Theta^{(t+1)} = \mathrm{argmax}_{\Theta} \langle \log p(\vec{y}, \vec{s} \mid \Theta) \rangle_{q(\vec{s}|\Theta^{(t)})}. \tag{4}$$

To keep the derivation focused, we present a simple derivation of the update equations only for a single element of W. The other parameters are similarly derived and are not covered here. For pedagogical purposes we first derive an update equation *without* a max rule, then we show how this rule should be modified when the max rule is used. Assuming the data $y^{(n)}$ is distributed as follows:

$$y^{(n)} = w s^{(n)} + \varepsilon \tag{5}$$

where $\varepsilon \sim \mathcal{N}(\mu = 0; \sigma^2)$. for w. This gives the conditional probability as:

$$p(y^{(n)} \mid s^{(n)}, w) = \frac{1}{\sigma\sqrt{2\pi}} \exp\left(-\frac{1}{2}\left(\frac{y^{(n)} - w s^{(n)}}{\sigma}\right)^2\right) \tag{6}$$

In log space this is a quadratic function:

$$\log p(y^{(n)} \mid s^{(n)}, w) = c - \log\sigma - \frac{1}{2}\left(\frac{y^{(n)} - w s^{(n)}}{\sigma}\right)^2 \tag{7}$$

and is summed over all datapoints n. The maximum likelihood solution differentiates this sum with respect to w (this function is linear in σ and when differentiated σ can be discarded) to find the maximum:

$$\frac{d}{dw}\left[\sum_{n}\left(y^{(n)} - s^{(n)} w\right)^2\right] = 0. \tag{8}$$

From which the maximum is given by:

$$w = \frac{\sum_n s^{(n)} w^{(n)}}{\sum_n s^{(n)2}}. \tag{9}$$

However, we care about finding the ML solution for the max rule:

$$y^{(n)} = \max_h \left\{ W_h s_h^{(n)} \right\} + \varepsilon \tag{10}$$

If the new estimates of W_h do not change significantly then the simple derivation for w will apply to W_h, but only the data for which W_h is the maximum will be used. The data is going to vary over: the number of images N, the number of samples per image K, and we will estimate W_{hd} per latent dimension h and observed dimension (or pixel) d. This leads to:

$$W_{hd} = \frac{\sum_n^N \sum_k^K \delta(\text{h is max}) s_{hn}^{(k)} y_d^{(n)}}{\sum_n^N \sum_k^K \delta(\text{h is max})\ {s_{hn}^{(k)}}^2} \tag{11}$$

which corresponds to the results given in equation (9) of the main paper. δ(h is max) is used to identify the index for which $W_{hd} s_{hn}^k$ is the maximal cause of the data, if it is not the maximal cause, then $\delta(\cdot)$ returns 0, and the term does not contribute to the sum.

References

[1] Neal R, Hinton G. A View of the EM Algorithm that Justifies Incremental, Sparse, and other Variants. In: Jordan MI, editor. Learning in Graphical Models. Kluwer; 1998. .
