# Supplementary material for: Nonlinear Spike-And-Slab Sparse Coding for Interpretable Image Encoding
Source: PLoS One. 2015 May 8;10(5):e0124088. doi: 10.1371/journal.pone.0124088 (PMC4425358; doi:10.1371/journal.pone.0124088)
Supplement: S1 Results — The complete set of generative fields learned W learned in the experiments on natural image patches and a larger set of the learned prior activations are shown here. (PDF) [file pone.0124088.s002.pdf]

S2 Results

Experiments: Natural Image Patches

Here we show further results from the experiment on $N = 50,000$ preprocessed and channel split natural image patches of 16×16 pixel.

First we relate the generative fields that are learned from image patches to their corresponding receptive fields as measured in biological systems. In order to do so we carried out several reverse correlation experiments, with the aim of identifying the relationship between the generative fields and the reverse-correlation fields. To calculate the reverse correlation for a single latent variable we calculate the average activation for a particular image (since the code is sparse, most of the time activations will be zero). The images are then averaged together, weighted by the average activation.

In Figure 1**A** we show the generative fields that are learned with our method. Figure 1**B** shows the receptive fields obtained by estimating the first order linear mapping from input to hidden units. The mapping is estimated by combining the preprocessing (a linear mapping with a kernel for pseudo-whitening) with the mapping obtained by reverse correlation using preprocessed patches. As can be observed by comparing Figure 1**A** and **B**, the qualitative and quantitative shapes of generative and receptive fields are essentially equal. For the results in the main text in Figure 1 we used the receptive field estimates in Figure 1**B**. Please see the preliminary study [1] for more details and analysis of the model's application to response properties in primary visual cortex.

In Figure 2, we see a histogram of the latent activations of the 20 most often active dictionary elements, all of which follow a spike-and-slab distribution, consistent with our prior beliefs.

References

[1] Shelton J, Sterne P, Bornschein J, Sheikh AS, Lücke J. Why MCA? Nonlinear sparse coding with spike-and-slab prior for neurally plausible image encoding. Advances in Neural Information Processing Systems. 2012;25:2285–2293.

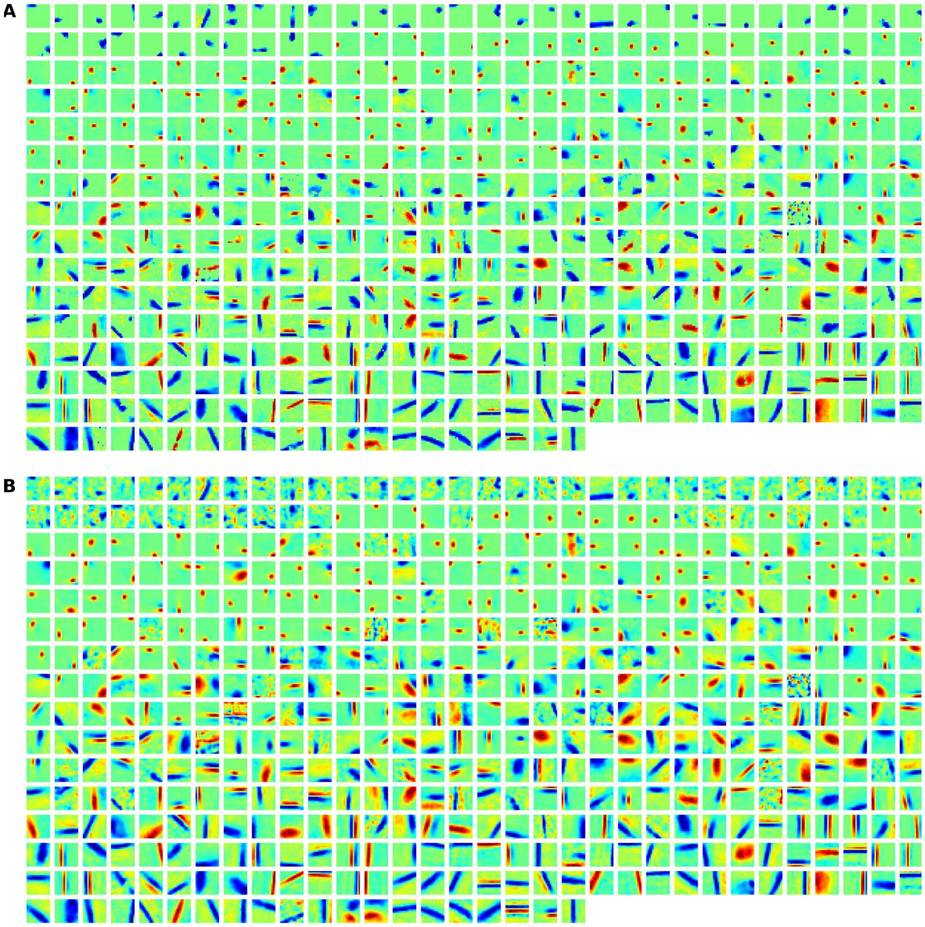

Figure 1: **A** Full set of $H = 500$ learned generative fields ($\vec{W}_h$). **B** Fields after reverse correlation with preprocessed input patches.

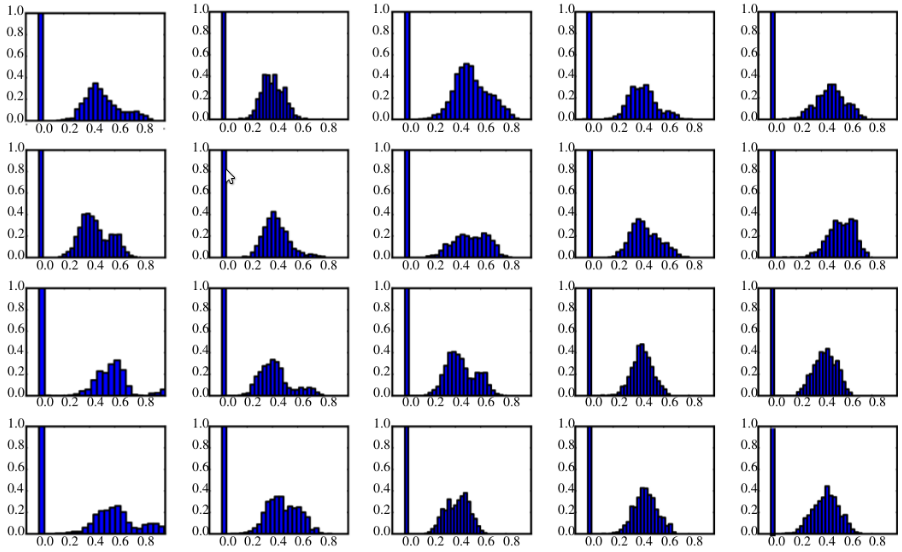

Figure 2: Histogram of the latent activations of the 20 most often active dictionary elements. This corresponds to the prior over latent units that we have assumed in our model, thus supporting the consistency of the model.
